# Molecular binding studies of anthocyanins with multiple antiviral activities against SARS-CoV-2

**DOI:** 10.1186/s42269-022-00786-0

**Published:** 2022-04-13

**Authors:** Precious Ayorinde Akinnusi, Samuel Olawale Olubode, Wasiu Adeboye Salaudeen

**Affiliations:** Department of Biochemistry, Adekunle Ajasin University, Akungba-Akoko, Nigeria

**Keywords:** SARS-CoV-2, Anthocyanins, Helicase, 3CL protease, RNA-dependent RNA polymerase, ACE-2, Molecular docking, Pharmacophore modeling, ADME/Tox

## Abstract

**Background:**

The search for ideal drugs with absolute antiviral activity against SARS-CoV-2 is still in place, and attention has been recently drawn to natural products. Several molecular targets have been identified as points of therapeutic intervention. The targets used in this study include SARS-CoV-2 helicase, spike protein, RNA-dependent RNA polymerase, main protease, and human ACE-2. An integrative computer-aided approach, which includes molecular docking, pharmacophore modeling, and pharmacokinetic profiling, was employed to identify anthocyanins with robust multiple antiviral activities against these SARS-CoV-2 targets.

**Result:**

Four anthocyanins (Delphinidin 3-O-glucosyl-glucoside, Cyanidin 3-O-glucosyl-rutinoside, Cyanidin 3-(p-coumaroyl)-diglucoside-5-glucoside), and Nasunin) with robust multiple inhibitory interactions were identified from a library of 118 anthocyanins using computer-aided techniques. These compounds exhibited very good binding affinity to the protein targets and moderate pharmacokinetic profiles. However, Cyanidin 3-O-glucosyl-rutinoside is reported to be the most suitable drug candidate with multiple antiviral effects against SARS-CoV-2 due to its good binding affinity to all five protein targets engaged in the study.

**Conclusions:**

The anthocyanins reported in this study exhibit robust binding affinities and strong inhibitory molecular interactions with the target proteins and could be well exploited as potential drug candidates with potent multiple antiviral effects against COVID-19.

## Background

Severe acute respiratory syndrome—coronavirus 2 (SARS-CoV-2)—is known to be a highly infectious betacoronavirus that affects humans (Lu et al. 2020). The SARS-CoV-2 outbreak has been declared as a Public Health Emergency of International Concern (PHEIC) since 2020, and efforts have been intensified to discover antiviral drugs with potent activity against SARS-CoV-2 (Burki 2020).

Many viral protein molecules encoded by the viral genome play crucial roles in the virulence, transmissibility, and replication of SARS-CoV-2. They include the main protease (also known as the SARS-CoV-2 main protease enzyme or 3C protease) which plays a critical role in the SARS-CoV-2 life cycle by aiding the process of viral replication and transcription, the helicase protein (also called nonstructural protein 13) which plays a huge role in viral replication, RNA-dependent RNA polymerase, and the receptor-binding domain (RBD) of S-protein which binds strongly to the human angiotensin-converting enzyme 2 (ACE2) receptors among others (Lan et al. 2020; Jin et al. 2020; Newman et al. 2021).

The identification and development of compounds from natural origin, particularly plants, into potential drug candidates have shown encouraging and reassuring prospects that these compounds, including polyphenols, may be employed as therapeutic interventions against viral diseases (Mohammadi Pour et al. 2019). The polyphenols are plant secondary metabolites that structurally have more than one phenolic ring and different structural elements that bind these rings to one another. Different compounds, including flavonoids, belong to this category of secondary metabolites (Fakhar et al. 2021). Flavonoids are in turn classified into four subclasses, namely anthocyanins, flavonols, flavones, and flavanones (Castro-Acosta et al. 2016). Anthocyanins are glycosides of flavonoid with anthocyanidin C6-C3C6 skeleton, also called flavylium (2-phenylchromenylium) ion (Smeriglio et al. 2016). Several anthocyanin derivatives have been identified, and only six major anthocyanin derivatives are extensively dispersed, and they include pelargonidin, delphinidin, petunidin, cyanidin, peonidin, and malvidin (Prior and Wu 2006). Recent studies have shown that anthocyanins are potent antiviral agents, notably against influenza virus, and have the potentials as antivirals against SARS-CoV-2 (Mohammadi Pour et al. 2019; Fakhar et al. 2021; Messaoudi et al. 2021). Studies have also identified flavonoid derivatives with the same basic structure as anthocyanins having potential antiviral activity against SARS-CoV-2 main protease (Jo et al. 2020). However, the studies were restricted to one or two molecular targets. This study aims to identify anthocyanins with multiple effects against SARS-CoV-2.

Herein, a computational approach was adopted to investigate the therapeutic potentials of various anthocyanins against COVID-19. The targets include SARS-CoV-2 3CL protease, RNA-dependent RNA polymerase (RdRp), spike protein receptor-binding domain, helicase, and human ACE-2. An integrated computational approach was adopted to identify anthocyanins with potential multiple antiviral activities. The workflow includes molecular docking, ADME/Tox screening, and 3D pharmacophore modeling.

## Methods

### Protein targets and ligand structures

The SMILES structures of 118 anthocyanin derivatives were extracted from PhytoHub online database and converted to SDF using Marvin sketch graphical user interface. The compounds were imported into Maestro 11.5 on Schrodinger suite and prepared using the LigPrep functional tool. The 3D crystal structures of the target proteins, SARS-CoV-2 3CL protease (PDB ID: 7JT7), RNA-dependent RNA polymerase (PDB ID: 6M71), spike protein (PDB ID: 6LZG), helicase (PDB ID: 7NNG), and human ACE-2 (PDB ID: 6LZG) were also retrieved from an online repository (Protein Data Bank) and prepared subsequently for docking.

### Molecular docking

The molecular docking procedure was carried out using the Glide script (Friesner et al. 2004) on Maestro 11.5. The compounds were docked into the prepared grid of the protein targets to identify compounds with potent inhibitory interactions with the proteins (Halgren 2009). The molecular docking procedure was initiated with the enzyme treated as a rigid body, while the ligand’s rotatable bonds were made to be flexible. The three levels of precision were employed in docking the compounds to the grids of the target proteins. Initially, high-throughput virtual screening (HTVS) was used to screen the compounds; subsequently, standard precision (SP) was employed to screen the highest-scoring compounds obtained from HTVS analysis. Finally, the more rigorous extra precision (XP) screened and scored the compounds. The top-scoring compounds were selected for post-docking analyses.

### ADMET analysis

The ADME/Tox properties of the lead compounds, which include absorption, distribution, metabolism, excretion, and toxicity, were profiled using SwissADME and ProTox-II online servers. The pharmacological properties predicted include lipophilicity, water solubility, drug-likeness, bioavailability score, blood–brain barrier permeation, reaction with cytochrome p450 isoforms, LD50, carcinogenicity, and possible hepatotoxicity.

### Pharmacophore modeling

The receptor–ligand complexes of the lead compounds were analyzed, and a hypothesis (E-pharmacophore) was generated using the phase interface of Schrodinger suite to highlight the major properties that actively contribute to the characteristic binding of the lead ligands to the active sites of the target proteins.

## Results

One hundred and eighteen anthocyanins were analyzed to determine their potential inhibitory activity against five SARS-CoV-2 targets. Eight compounds with multiple binding affinities to the target proteins were identified. The docking scores of the top-scoring compounds of each target are presented in Table 1. The docking scores demonstrate the binding energy exhibited by the compounds in complex with the molecular targets analyzed. **C1** (Delphinidin 3-O-glucosyl-glucoside) showed the highest binding energy when complexed with 3Cl protease. Similarly, **C2** (Cyanidin 3-O-glucosyl-rutinoside) had the highest docking score against the helicase protein and **C3** (Cyanidin 3-(p-coumaroyl)-diglucoside-5-glucoside) against RNA-dependent RNA polymerase. Furthermore, **C3** (Cyanidin 3-(p-coumaroyl)-diglucoside-5-glucoside) and **C8 (**Nasunin) had the highest docking scores in complex with SARS-CoV-2 spike protein and human ACE-2, respectively.

**Table 1:** Docking scores of top-scoring anthocyanins against SARS-CoV-2 targets

Compounds	SARS-CoV-2 3Cl pro	SARS-CoV-2 Helicase	SARS-CoV-2 RNA-dependent RNA polymerase	SARS-CoV-2 Spike protein	HUMAN ACE-2
C1	− 12.77	− 10.75	− 10.85	− 6.76	− 12.18
C2	− 10.67	− 10.93	− 10.10	− 10.07	− 13.26
C3	− 10.54	− 11.58	− 13.82	− 6.10	− 13.67
C4	− 7.51	− 11.72	− 10.22	− 7.32	− 11.42
C5	− 10.00	− 7.67	− 12.73	− 4.19	− 12.07
C6	− 7.69	− 6.17	− 8.91	− 7.61	− 13.56
C7	− 8.22	− 7.59	− 9.12	− 7.45	− 12.25
C8	− 11.30	− 9.53	− 10.88	− 6.97	− 13.90
Hydroxychloroquine	− 6.19	− 4.57	− 1.47	− 1.82	− 6.31
Remdesivir	− 5.88	− 4.88	− 6.67	− 2.16	− 5.70

**C1** = Delphinidin 3-O-glucosyl-glucoside, **C2** = Cyanidin 3-O-glucosyl-rutinoside, **C3** = Cyanidin 3-(p-coumaroyl)-diglucoside-5-glucoside, **C4** = Cyanidin 3-O-xylosyl-rutinoside, **C5** = Cyanidin 3-(sinapoyl)-diglucoside-5-glucoside, **C6** = Petunidin 3-O-rutinoside, **C7** = Malvidin 3-O-(6''-caffeoyl-glucoside), **C8 = **Nasunin

The physicochemical properties of the identified compounds are presented in Table 2. The predicted log *p* value which represents the lipophilicity ranged from − 3.68 to 0.42 with C7 having the highest value and C2, the lowest. The consensus log p value is the arithmetic mean of five different predictive models of lipophilicity, namely iLOGP, XLOGP3, WLOGP, MLOGP and Silicos-IT log P. C7, which had the highest value, has the highest probability of gastrointestinal absorption. Consequently, the predicted Silicos-IT log SW which measures the water solubility of the compounds ranged from -3.23 to 2.12 with C2 being the most water soluble and C7, the least soluble. Water solubility contributes substantially to the movement of small molecular weight compounds in systemic circulation.

**Table 2:** SWISSADME-predicted lipophilicity (Log P) and water solubility (Log Sw)

Compounds	Consensus Log P	Silicos-IT LogSw	Silicos-IT class
C1	− 3.18	1.46	Soluble
C2	− 3.68	2.12	Soluble
C3	− 2.85	0.65	Soluble
C4	− 3.53	1.68	Soluble
C5	− 2.81	0.5	Soluble
C6	− 3.23	0.28	Soluble
C7	0.42	− 3.23	Soluble
C8	− 2.59	1.05	Soluble

**C1** = Delphinidin 3-O-glucosyl-glucoside, **C2** = Cyanidin 3-O-glucosyl-rutinoside, **C3** = Cyanidin 3-(p-coumaroyl)-diglucoside-5-glucoside), **C4** = Cyanidin 3-O-xylosyl-rutinoside, **C5** = Cyanidin 3-(sinapoyl)-diglucoside-5-glucoside, **C6** = Petunidin 3-O-rutinoside, **C7** = Malvidin 3-O-(6''-caffeoyl-glucoside), **C8 = **Nasunin

The amino acid interaction of the top-scoring compounds with the active sites of the target proteins are presented in Table 3. Covalent hydrogen bond is the major form of interaction observed in the characteristic binding of the compounds to each protein target.

**Table 3:**
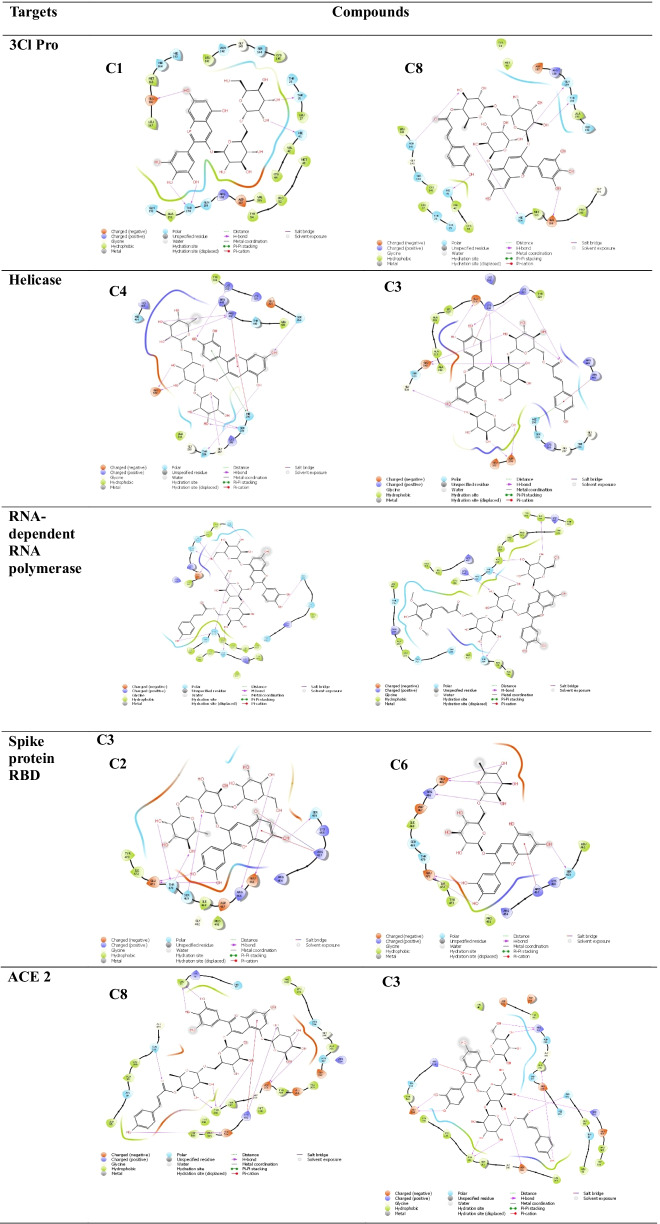
2D Amino acid interaction of the top-scoring compounds

The drug-likeness and bioavailability scores of the top-scoring compounds are presented in Table 4. All the compounds have a bioavailability score of 0.17, and all violated 3 of the Lipinski rule-based filter of drug-likeness. Similarly, all the test compounds violated 1 of the Veber rule-based filters except C5 and C8 with 2 violations. Drug-likeness and bioavailability score predicts the likelihood of a compound being a drug candidate.

**Table 4:** Drug-likeness and bioavailability

Compounds	Lipinski #violations	Veber #violations	Bioavailability score
C1	3	1	0.17
C2	3	1	0.17
C3	3	1	0.17
C4	3	1	0.17
C5	3	2	0.17
C6	3	1	0.17
C7	3	1	0.17
C8	3	2	0.17

The SwissADME predicted pharmacokinetic profiles of the compounds are presented in Table 5. All the compounds do not have a structural orientation that would enable them to permeate the blood–brain barrier. C2, C3, C4, and C5 are predicted to be substrates of permeability glycoprotein (P-gp), while C1, C6, C7, and C8 are non-substrate.

**Table 5:** Predicted pharmacokinetic properties of test compounds

Compounds	BBB permeant	Pgp substrate	CYP1A2 inhibitor	CYP2C19 inhibitor	CYP2C9 inhibitor	CYP2D6 inhibitor	CYP3A4 inhibitor	Log Kp (cm/s)
C1	No	No	No	No	No	No	No	− 11.8
C2	No	Yes	No	No	No	No	No	− 13.12
C3	No	Yes	No	No	No	No	No	− 12.98
C4	No	Yes	No	No	No	No	No	− 13.73
C5	No	Yes	No	No	No	No	No	− 13.39
C6	No	No	No	No	No	No	No	− 11.84
C7	No	No	No	No	No	No	No	− 9
C8	No	No	No	No	No	No	No	− 13.12

**Fig. 1:**
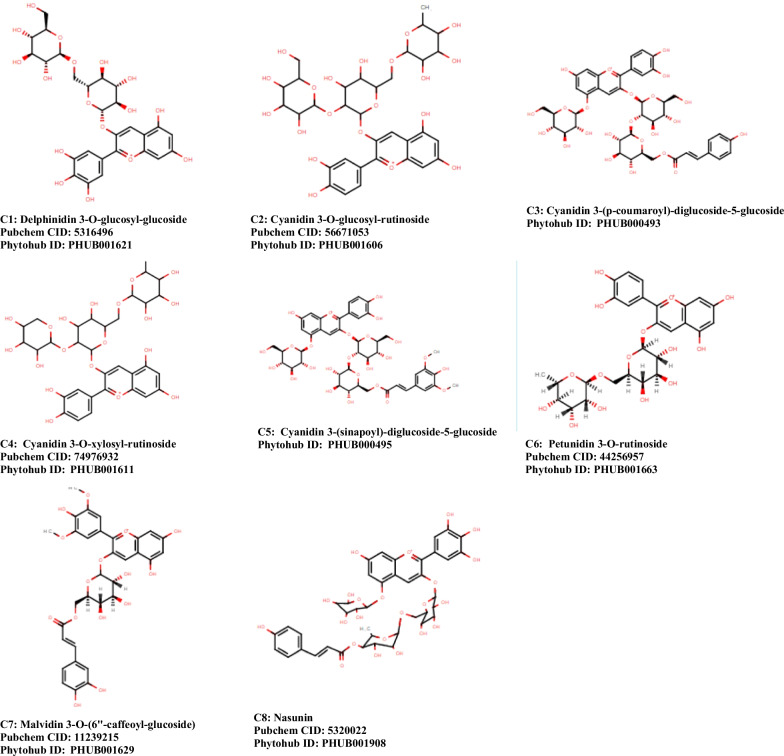
Structures of reported anthocyanins

The ProTox-II-predicted toxicity profiles of the compounds are presented in Table 6. All the compounds have the same toxicity profile, an LD50 of 5000 mg/kg BW, and are not carcinogenic neither do they have a probability to cause harm to the liver (Fig. 1).

**Table 6:** ProTox-II toxicity prediction

Compounds	LD50 (mg/kg)	Toxicity class	Hepatotoxicity	Carcinogenicity
C1	5000	5	–	–
C2	5000	5	–	–
C3	5000	5	–	–
C4	5000	5	–	–
C5	5000	5	–	–
C6	5000	5	–	–
C7	5000	5	–	–
C8	5000	5	–	–

**C1** = Delphinidin 3-O-glucosyl-glucoside, **C2** = Cyanidin 3-O-glucosyl-rutinoside, **C3** = Cyanidin 3-(p-coumaroyl)-diglucoside-5-glucoside), **C4** = Cyanidin 3-O-xylosyl-rutinoside, **C5** = Cyanidin 3-(sinapoyl)-diglucoside-5-glucoside, **C6** = Petunidin 3-O-rutinoside, **C7** = Malvidin 3-O-(6''-caffeoyl-glucoside), **C8 = **Nasunin

**Fig. 2:**
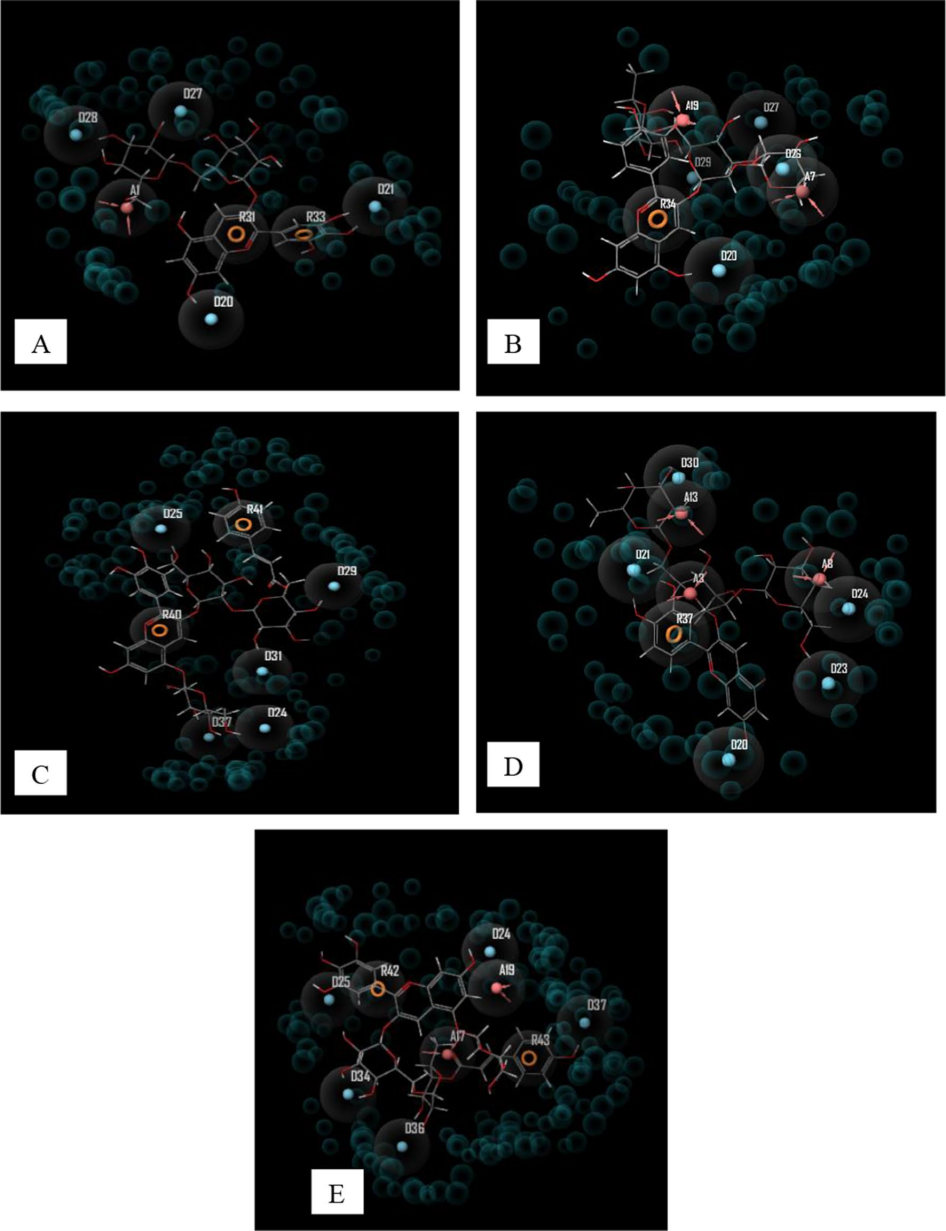
Pharmacophore model of top-scoring compounds. A = C1-3Cl protease complex, B = C4-Helicase complex, C = C3-RNA-dependent RNA polymerase complex, D = C2-Spike protein RBD complex, E = C8-Ace 2 complex

Figure 2 shows the pharmacophore model of the top-scoring compounds in each protein target. The model shows that aromatic ring and hydrogen bond (donor and acceptor) are the major forms of interactions that contribute to the binding of the compounds to the protein targets.

## Pharmacophore model

### Discussion

The global spread of SARS-CoV-2 requires urgent and novel therapeutic discoveries given the high failure rate of traditional drug discovery methods (Mirabelli et al. 2021). Attentions have been constantly drawn to natural compounds due to their relatively lower side effects (Lin et al. 2017). In light of this, different anthocyanins have been previously shown to be good inhibitors of SARS-CoV-2 molecular targets (Fakhar et al. 2021; Messaoudi et al. 2021).

Eight anthocyanins with impressive binding data were identified from 118 anthocyanins screened, and they were found to show robust binding affinity to the protein targets. Hydrogen bonding is the major form of interaction observed in the binding data. In all categories, the identified anthocyanins have higher binding affinity than the standard drugs: remdesivir and hydroxychloroquine. C1, C2, C3, C5, and C8 showed impressive binding affinity to SARS-CoV-2 3CL protease. The protein functions in the maturation of viral polyprotein and is essential for the completion of SARS-CoV-2 life cycle (Kan et al. 2005). Therefore, inhibiting this protein is a proven therapeutic option in curbing coronavirus disease. All the compounds exhibited good binding affinity to 3CL protease but C1, C2, C3, C5, and C5 have greater binding than the other compounds. Similarly, C1, C2, C3, C4, and C8 showed excellent binding affinity to SARS-CoV-2 helicase. The helicase protein, also called non-structural protein 13 (NSP 13), has been identified as a target for antivirals due to its role in viral replication (Newman et al. 2021). Of all compounds tested, C4 had the highest docking score (− 11.72) and the pharmacophore model showed that hydrogen bond and aromatic rings contribute substantially to its binding to NSP13.

All the test compounds (Fig. 1) exhibited good molecular binding affinity to RNA-dependent RNA polymerase (RdRp) also named NSP 12. The docking scores of the compound ranged from − 12.73 to − 8.91 with C3 having the highest binding affinity to the protein. C3 interacted with seven amino acids in the active site of NSP12 (THR319, SER255, ASP390, THR393, THR394, SER397, and ASN459).

Only C2 (− 10.07) showed a good binding affinity to the receptor-binding domain of the viral spike protein required for viral entry into the host’s cell. Finally, all the compounds exhibited robust binding affinity (− 11.42 to − 13.9) to the fifth protein target: human angiotensin-converting enzyme 2 (ACE2) which is the receptor for SARS-CoV-2 entry into the human host.

**Fig. 3:**
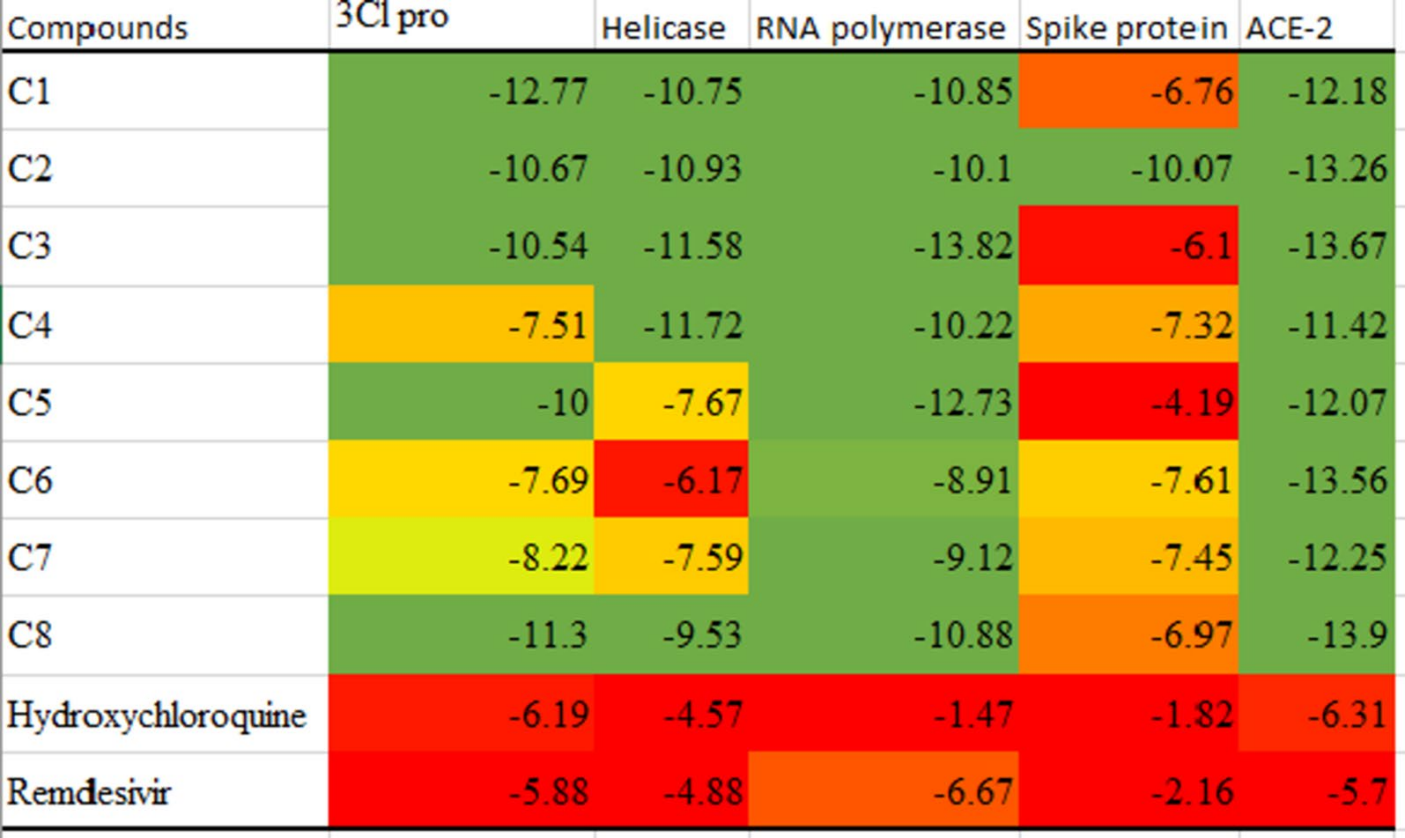
Heat map of the docking scores of the top-scoring compounds

Considering the heat map of the docking scores (Fig. 3), it can be adjudged that C2 is the most suitable multitarget antiviral agent against SARS-CoV-2 among the compounds reported. Similarly, C1, C3, and C8 also showed promising inhibitory potential against the target proteins.

### ADMET profile

The predicted value of log P measures the lipophilicity of the compounds. For better accuracy, the arithmetic mean of five different models of the partition coefficient of n-octanol to water was adopted as the log P in this study. An oral drug candidate must be sufficiently lipophilic to enable it to cross the intestine into the systemic circulation. C7 had the highest (0.42) log P value and is predicted to be the most lipophilic of all compounds tested. The value of Silicos-IT Log SW as predicted by SwissADME represents the degree of solubility in water. C2 was found to be the most water-soluble. Drugs are transported to the cells that need them through the hydrophilic systemic circulation; therefore, a drug candidate must be sufficiently hydrophilic to aid its transport in the systemic circulation.

The pharmacokinetic screening of the compounds showed that they all have a bioavailability sore of 0.17. Abbot Bioavailability Score is the likelihood of a compound having greater than 10% bioavailability in rats or measurable Caco-2 permeability (Martin 2005).

All the compounds are predicted to be non-substrates of the CYP isoforms engaged and would not elicit a drug–drug interaction. Cytochrome P450 is a family of highly similar enzymes that play a big role in the metabolism and excretion of various compounds. Studies have suggested that about 50–90% of biologically active compounds are substrates of five isoforms of the superfamily (CYP1A2, CYP2C19, CYP2C9, CYP2D6, and CYP3A4) (Diana et al. 2017), and inhibition of the activity of these enzymes can cause a drug–drug response (Huang et al. 2008).

## Conclusions

Delphinidin 3-O-glucosyl-glucoside, Cyanidin 3-O-glucosyl-rutinoside, Cyanidin 3-(p-coumaroyl)-diglucoside-5-glucoside), and Nasunin exhibit robust binding data to five SARS-CoV-2 molecular targets; therefore, these compounds are recommended for further analyses as they could be explored as potential antiviral agents with multiple targets against SARS-CoV-2.

## Data Availability

The datasets generated are available from the corresponding author on request.

## Abbreviations

ACE: Angiotensin-converting enzyme
CoV: Coronavirus
CYP: Cytochrome P450
NSP: Non-structural protein
P-gp: Permeability glycoprotein
RBD: Receptor-binding domain
RdRp: RNA-dependent RNA polymerase
SARS: Severe acute respiratory syndrome
